# Effekt von Behandlungsverzögerungen auf die Mortalität von Krebserkrankungen: systematische Übersicht und Metaanalyse

**DOI:** 10.1007/s43472-021-00041-3

**Published:** 2021-06-18

**Authors:** Philip C. Müller, Matthias Turina

**Affiliations:** Klinik für Viszeral- und Transplantationschirurgie, Universitätsspital Zürich, Rämistrasse 100, 8091 Zürich, Schweiz

## Originalpublikation

Hanna TP, King WD, Thibodeau S et al (2020) Mortality due to cancer treatment delay: systematic review and meta-analysis. BMJ 371:m4087. 10.1136/bmj.m4087.

## Hintergrund der Studie.

Verzögerungen in der kurativen Behandlung von Krebserkrankungen können negative Auswirkungen auf den Verlauf der Krankheit haben. In Vorarbeiten konnte bereits ein Zusammenhang zwischen Behandlungsverzögerung und Mortalität von Krebserkrankungen gezeigt werden. Diese Vorarbeiten sind jedoch in ihrer Aussagekraft durch uneinheitliche Definition der Behandlungsverzögerung limitiert. Durch die COVID-19-Pandemie hat allerdings die Fragestellung nach dem Einfluss einer verzögerten Behandlung von Krebserkrankungen deutlich an Brisanz gewonnen, so fand doch in den meisten Kliniken bzw. Gesundheitssystemen eine Umstrukturierung der Ressourcen statt. Durch den Einsatz von Ressourcen zur Bewältigung der COVID-19-Pandemie wurde vielerorts elektive Behandlungen wie die chirurgische oder onkologische Therapie (Radio- und Chemotherapie) von Krebserkrankungen verzögert. Gerade in Zeiten von knappen Ressourcen im Gesundheitswesen ist das Verständnis zwischen dem Zusammenhang von Behandlungsverzögerungen und damit einhergehenden negativen Auswirkungen essenziell für die Etablierung von klinischen Behandlungspfaden und gerechter Allokation der knappen Ressourcen in unserem Gesundheitssystem.

## Ziel der Studie.

Anhand der höchsten Evidenz Evaluation des Zusammenhangs zwischen Behandlungsverzögerung von kurativen Therapien und Mortalitätsentwicklung bei 7 unterschiedlichen Krebserkrankungen. Die Daten sollen sowohl eine Priorisierung von Behandlungen als auch die Optimierung von Behandlungspfaden in Zentren mit Spezialisierung auf die Therapie von Krebserkrankungen ermöglichen.

## Methoden.

Patienten mit einer kurativ-intendierten chirurgischen Therapie mit neoadjuvanter bzw. adjuvanter Therapie als auch Patienten mit kurativer Systemtherapie oder Radiotherapie wurden eingeschlossen. In der Studie berücksichtigt wurden Patienten mit Blasen‑, Brust‑, Kolon, Rektum‑, Lungen‑, Zervix- und HNO-Karzinomen, wobei die eingeschlossenen Krebserkrankungen 44 % der Krebserkrankungen abdecken. In dem systematischen Review wurden Studien berücksichtigt, die zwischen Januar 2000 und April 2020 publiziert wurden. Als Behandlungsverzögerung wurde die Zeit zwischen Diagnose und erster Behandlung (bei chirurgischer Therapie oder Bestrahlung) bzw. zwischen chirurgischer Therapie und adjuvanter Therapie definiert. Bei neoadjuvantem Therapiekonzept wurde die Zeit von der Diagnosestellung bis zum Start der neoadjuvanten Therapie bzw. die Zeit vom Ende der neoadjuvanten Therapie bis zur Operation als Therapieverzögerung definiert. Primärer Endpunkt der Studie war die Hazard-Ratio für das Gesamtüberleben, aufgeteilt in 4‑Wochen-Abständen. Hierbei errechnet sich die Hazard-Ratio aus dem Vergleich des Mortalitätsrisikos von Patienten mit Behandlungsverzögerung und solchen ohne.

## Ergebnisse.

In das Review wurden 34 Studien mit 1.272.681 Patienten eingeschlossen, wobei alle Studien ein retrospektives Design hatten. Die Assoziation zwischen verzögerter Behandlung und erhöhter Mortalität war statistisch signifikant für 13 der 17 Therapieindikationen. In der Metaanalyse waren die Ergebnisse konsistent für die chirurgische Behandlungen mit einer Erhöhung des 4‑Wochen-Mortalitätsrisikos bei Verzögerung der Behandlung mit einer Hazard-Ratio von 1,06–1,08. Dies entspricht einer 6–8 %igen Erhöhung der Mortalität für jede Verzögerung einer kurativen Operation um 4 Wochen! Bei Kolonkarzinomen war die Hazard-Ratio bei 1,06 (1,01–1,12), entsprechend einer 6 %igen Erhöhung der Mortalität für jede 4‑wöchige Verzögerung der Behandlung. Der Effekt variierte bei adjuvanten und neoadjuvanten Behandlungen mit einer Hazard-Ratio von 1,01–1,28 deutlich mehr. So fand sich z. B. für die Verzögerung der adjuvanten Therapie bei Kolon- und Rektumkarzinomen ein Anstieg der Mortalität um 13 % (9–17 %) für jedes Intervall von 4 Wochen. Keine qualitativ hochwertigen Studien untersuchten den Effekt der Behandlungsverzögerung bei neoadjuvanter Therapie beim Rektumkarzinom. Die Analyse von Daten bei kurativer Radiotherapie zeigte vor allem bei HNO- (1,09; 1,05–1,14) und Zervixkarzinomen (1,23; 1,00–1,50) einen Effekt bei Verzögerungen.

## Kommentar zur Studie

In dieser grossen systematischen Übersicht mit Metaanalyse konnte ein deutlicher Zusammenhang zwischen verzögerter kurativer Behandlung und Anstieg der Mortalität gezeigt werden. Dies konnte für 7 Tumortypen (Blasen‑, Brust‑, Kolon, Rektum- Lungen‑, Zervix- und HNO-Karzinome) sowie für 3 Behandlungsmodalitäten (Chirurgie, systemische Therapie und Radiotherapie) gezeigt werden. Bei Verzögerung einer chirurgischen Behandlung nimmt die Mortalität mit jedem Intervall von 4 Wochen Verzögerung um 6–8 % zu. Bei systemischen Therapien war die Zunahme mit 9–13 % sogar noch grösser. Hierbei ist zu beachten, dass Verzögerungen über die 4 Wochen hinaus zu einer weiteren Zunahme der Mortalität führen. Die Autoren zeigen auf, dass die Minimierung der Zeit zwischen Diagnose und kurativer Therapie ähnlich vorteilhafte Effekte hat wie gewisse neue systemische Therapien. Wichtig für die Interpretation dieser Daten ist, dass die Verzögerung multifaktorielle Gründe haben kann: So können sowohl Patientenfaktoren (präoperative Abklärungen, postoperative Komplikationen), krankheitsspezifische Faktoren oder systembedinge Faktoren (Zeit bis Vorstellung beim Spezialist, Operationskapazitäten, Behandlungspfade) einen Einfluss auf den Zeitpunkt des Therapiebeginns haben. Die Autoren hatten zum Ziel, gerade im Hinblick auf die COVID-19-Pandemie auf möglichst geringe Verzögerungen durch systemische Faktoren hinzuweisen. Im Hinblick auf das kolorektale Karzinom zeigt sich anhand von Daten in Deutschland in der COVID-19-Pandemie ein deutlicher Rückgang der Vorsorgekoloskopien von 75 % [1], was eine entsprechende Verzögerung der Diagnostik mit sich bringen könnte. Ähnliche Daten finden sich in Amerika, wobei zu Beginn der Pandemie im April 2020 die Screeninguntersuchungen für Kolonkarzinome (− 74 %) und auch die Biopsien von Kolonkarzinomen (− 79 %) richtiggehend einbrachen [2]. Die Empfehlungen der „Foederatio Medicorum Helveticorum“ (FMH) gehen beim „Behandlungspfad-Schema Kolorektalkarzinom“ nicht auf zeitliche Abläufe ein bzw. definieren keine Soll-Zeit zwischen Diagnostik und kurativer Therapie [3]. Auch bei der Orientierung an den deutschen S3-Leitlinien Kolorektales Karzinom wird einzig erwähnt, dass die adjuvante Chemotherapie beim Kolonkarzinom postoperativ baldmöglichst eingeleitet werden sollte (Evidenzlevel 3b). Zusammenfassend konnte die Studie eine deutliche Erhöhung der Mortalität bei Behandlungsverzögerungen zeigen. Hierbei ist zu beachten, dass selbst Verzögerungen von 4 Wochen einen relevanten Einfluss auf die Sterblichkeit haben, was in künftigen Diskussionen um Ressourcenallokation bei externen Ereignissen wie der COVID-Pandemie berücksichtigt werden sollte.
